# Transfer learning with deep convolutional neural network for liver steatosis assessment in ultrasound images

**DOI:** 10.1007/s11548-018-1843-2

**Published:** 2018-08-09

**Authors:** Michał Byra, Grzegorz Styczynski, Cezary Szmigielski, Piotr Kalinowski, Łukasz Michałowski, Rafał Paluszkiewicz, Bogna Ziarkiewicz-Wróblewska, Krzysztof Zieniewicz, Piotr Sobieraj, Andrzej Nowicki

**Affiliations:** 1 Department of Ultrasound, Institute of Fundamental Technological Research, Polish Academy of Sciences, Pawińskiego 5B, 02-106 Warsaw, Poland; 2 Department of Internal Medicine, Hypertension and Vascular Diseases, Medical University of Warsaw, Warsaw, Poland; 3 Department of General, Transplant and Liver Surgery, Medical University of Warsaw, Warsaw, Poland; 4 Department of Pathology, Center for Biostructure Research, Medical University of Warsaw, Warsaw, Poland

**Keywords:** Nonalcoholic fatty liver disease, Ultrasound imaging, Deep learning, Convolutional neural networks, Hepatorenal index, Transfer learning

## Abstract

**Purpose:**

The nonalcoholic fatty liver disease is the most common liver abnormality. Up to date, liver biopsy is the reference standard for direct liver steatosis quantification in hepatic tissue samples. In this paper we propose a neural network-based approach for nonalcoholic fatty liver disease assessment in ultrasound.

**Methods:**

We used the Inception-ResNet-v2 deep convolutional neural network pre-trained on the ImageNet dataset to extract high-level features in liver B-mode ultrasound image sequences. The steatosis level of each liver was graded by wedge biopsy. The proposed approach was compared with the hepatorenal index technique and the gray-level co-occurrence matrix algorithm. After the feature extraction, we applied the support vector machine algorithm to classify images containing fatty liver. Based on liver biopsy, the fatty liver was defined to have more than 5% of hepatocytes with steatosis. Next, we used the features and the Lasso regression method to assess the steatosis level.

**Results:**

The area under the receiver operating characteristics curve obtained using the proposed approach was equal to 0.977, being higher than the one obtained with the hepatorenal index method, 0.959, and much higher than in the case of the gray-level co-occurrence matrix algorithm, 0.893. For regression the Spearman correlation coefficients between the steatosis level and the proposed approach, the hepatorenal index and the gray-level co-occurrence matrix algorithm were equal to 0.78, 0.80 and 0.39, respectively.

**Conclusions:**

The proposed approach may help the sonographers automatically diagnose the amount of fat in the liver. The presented approach is efficient and in comparison with other methods does not require the sonographers to select the region of interest.

## Introduction

The nonalcoholic fatty liver disease, diagnosed in a large number of obese patients, is the most common liver abnormality [1]. It is defined as the accumulation of fat in more than 5% of liver cells. This disease is associated with increased risk of hepatic cirrhosis and hepatocellular carcinoma, but it is also influencing higher cardiovascular morbidity and mortality in affected patients [2, 3]. Liver biopsy is the reference standard for direct liver steatosis quantification in hepatic tissue samples [4]. However, biopsy is a costly and invasive procedure that carries a high risk of serious complications, commonly including pain, bleeding and in rare cases, death [4]. Therefore, liver biopsy is not considered to be an easy, optimal way to assess and follow-up the progress of common liver diseases. Noninvasive liver imaging methods such as computed tomography, magnetic resonance imaging or ultrasound (US) have been intensively investigated [5]. US may be the preferred modality for screening liver steatosis because of its non-invasiveness, low cost and wide availability.

Up to date various approaches have been proposed to assess the level of steatosis in liver using US [6]. Among them, the hepatorenal sonographic index (HI) is considered to be highly efficient and simple [7, 8]. The HI method is based on comparison of the liver echogenicity to that of the right kidney cortex. Normal liver and renal tissues show similar echogenicity. However, in the presence of steatosis, the liver tissue brightness is higher than the kidney brightness. The ultrasound-based diagnostic results may depend on skills and experience of physicians performing the examination, type of ultrasound machine and even on US image settings [9, 10]. This operator dependence makes the comparison of results difficult and limits wider practical application of this important imaging technique. Another approach to liver steatosis assessment employs texture analysis. According to the review paper on liver image analysis [6], the gray-level co-occurrence matrix (GLCM) algorithm is the most frequently used method for liver disease characterization [11]. GLCMs provide useful information about spatial gray-level dependencies in an image. Texture patterns of US images arise from the interference of backscattered US waves on tissue microstructures. The GLCM-based approaches to liver steatosis classification using US images have been proposed in several papers [12–15].

Nowadays new algorithms for image analysis are intensively studied, including deep learning. These machine learning methods let the computers automatically develop useful features for classification. The usefulness of convolutional neural networks (CNNs) has been reported in solving various medical image analysis problems [16, 17]. CNNs transform input images with convolutional filters into a single decision variable as an output that usually indicates the input image label. However, to successfully train a CNN, usually a large amount of input data are required. This issue limits the practical applications of deep models in medical image analysis, since the available medical image datasets are usually small. Therefore, as a solution, various transfer learning techniques have been proposed [18]. Instead of building a completely new model from scratch, it is possible to use a model developed for another problem. The usefulness of a pre-trained model depends on its ability to adjust to images outside the original training dataset. In the case of medical image analysis, the implementation of transfer learning techniques has been reported in several papers [19–22].

The aim of this paper is to develop a deep learning model for steatosis level assessment based on US liver B-mode images and to compare it with the HI and the GLCM techniques. The US data analyzed in this study were collected from severely obese patients evaluated before bariatric surgery. We used a pre-trained CNN to extract features based on B-mode images. Next, using the neural features, we employed the support vector machine (SVM) algorithm to classify images containing fatty liver. Aside of fatty liver classification, it is clinically relevant to quantify the grade of liver steatosis. For this task, we used the extracted features and the Lasso regression method. In both cases, liver biopsy results served as a reference. The performance of the proposed approach was compared with the HI and the GLCM methods.

This paper is organized in the following way. First, we describe the patient group and the data acquisition routines. It is presented how to calculate the HI- and the GLCM-related features using liver US images. Next, our deep learning solution to fatty liver assessment is described. We show how to apply the transfer learning to extract CNN-based features using B-mode liver images. Next, we employ the CNN- and the GLCM-based features to perform fatty liver disease classification and to assess the level of steatosis. Results are presented and evaluated. Finally, we discuss the advantages and disadvantages of the applied methods.

## Materials and methods

### Clinical dataset

**Fig. 1:**
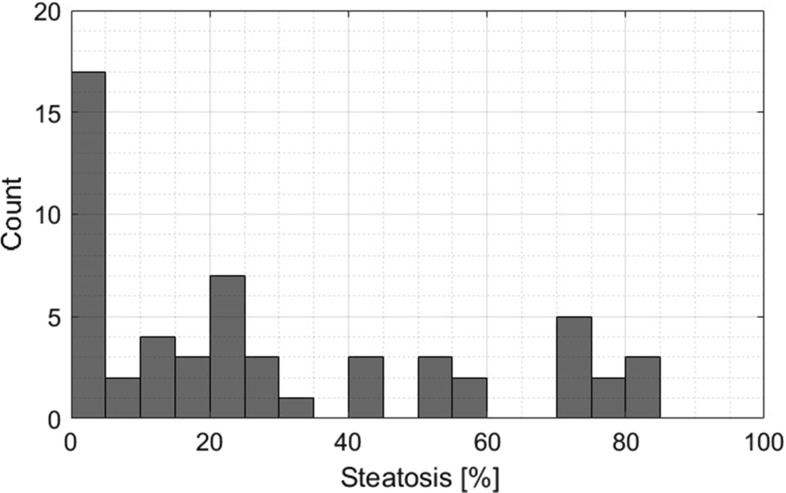
Histogram of steatosis level across the population of patients in the study group

Our study involved 55 severely obese patients (mean age 40.1 ± 9.1, mean BMI 45.9 ± 5.6, 20% of males) admitted for bariatric surgery (laparoscopic sleeve gastrectomy). The ultrasound data were acquired in the Department of Internal Medicine, Hypertension and Vascular Diseases, Medical University of Warsaw, Poland, during preoperative cardiac echocardiographic evaluation, 1–2 days before the surgery. The study was approved by the Ethical Committee at the Medical University of Warsaw, and all patients gave informed consent for echocardiography and abdominal ultrasound examination. Each patient underwent a wedge liver biopsy during the bariatric surgery as a part of the routine protocol implemented at the Department of General, Transplant and Liver Surgery, Medical University of Warsaw, Poland [23]. Tissue sample was extracted from the subcapsular part of the left liver lobe. The histopathological assessment was performed by a single pathologist following the recommendations of the Clinical Research Network [24]. The level of steatosis was defined based on the percentage of hepatocytes with fatty infiltration. The fatty liver was defined to have more than 5% hepatocytes with steatosis. The number of patients with fatty liver was equal to 38. The steatosis level distribution across the population of patients is depicted in Fig. 1.

**Fig. 2:**
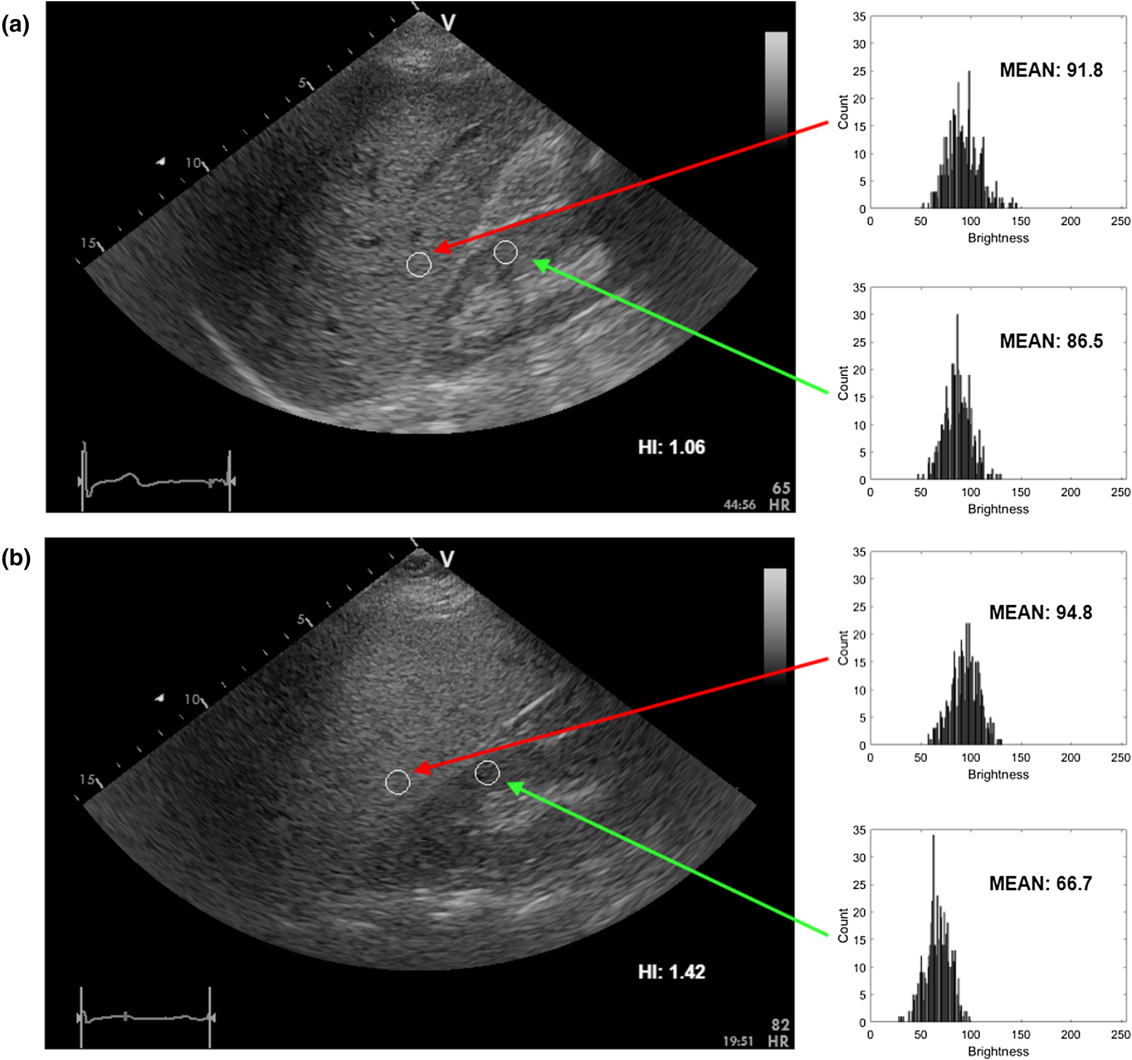
Liver B-mode images and the ROIs selected for HI calculation, **a** steatosis level of 3% and **b** 25%, respectively

The ultrasound data were acquired using the GE Vivid E9 Ultrasound System (GE Healthcare INC, Horten, Norway) equipped with a sector probe operating at 2.5 MHz. The default general abdominal preset with harmonic imaging was used. The resolution of B-mode images was equal to 434 × 636 (pixel size of 0.373 mm × 0.373 mm), see Fig. 2. For each patient, a sequence of B-mode images, corresponding to one heart beat, was acquired and stored on the workstation (EchoPac PC software, GE Healthcare INC, Norway). The image loops were saved in DICOM format for further off-line processing. Due to motion, the speckle patterns and relative position of the liver and the kidney were slightly different across the images in each sequence. Moreover, the number of images in sequences was not constant. It depended, for example, on the number of focal zones and the scanner frame rate. For each sequence, ten consecutive images were used for further processing. Finally, the dataset contained 550 B-mode images. We decided to analyze image sequences rather than single B-mode images selected by the physician. It was a way of data augmentation, which enabled us to provide more diverse data to the models.

The dataset described above can be downloaded via the Zenodo repository (10.5281/zenodo.1009146). The dataset repository includes sequences of B-mode images and the biopsy results. The provided dataset could be useful for researchers interested in fatty liver imaging. It should be noted that during the acquisition of the data with the cardiac probe, we recorded the images with the kidney on the left side of the screen. For convenience of those researchers who are used to kidney on the right side of the image, we provide in Fig. 2 the example images following the standard convention. In the case of the dataset, the images were provided with the left sided kidney arrangement as recorded during the image acquisition.

### Hepatorenal index

The HI is defined as the ratio of average brightness level of the liver and the kidney cortex. Generally, the HI is expected to increase with the steatosis level. In our study, the HI was determined by a physician with experience in ultrasonography and echocardiography research acquisition [25]. The physician was blind to biopsy results. In the first step, a single scan frame from the B-mode sequence was selected by the physician. Next, two regions of interest (ROIs) corresponding to the liver and the kidney cortex were specified. The ROI selection is illustrated in Fig. 2. Care was taken to select liver and kidney ROI in the middle part of the image sector, side by side at the same depth. If infeasible due to suboptimal image quality, liver ROI was selected above kidney ROI with the shortest distance possible. The ROI was determined by using circular method with the radius of the circle equal to 5 mm. In each case, the ROI was as uniform as possible. Regions of non-uniform speckle pattern, vessels or ducts were omitted during the ROI selection procedure. The ratio between the average brightness levels in the ROIs was determined with Matlab software (MathWorks INC, USA) using histogram analysis, see Fig. 2.

### GLCM-based features

GLCM-based features were extracted following a similar approach proposed in [12–14]. The same liver ROIs were employed for analysis as in the case of the HI method. However, instead of the circular regions, we used square regions with side length of 10 mm. For each ROI nine different GLCMs were calculated considering angles between 0, 45 and 135, and path distances of 1, 2 and 3 [12]. Next, for each GLCM the following texture features were extracted: maximum probability, uniformity, entropy, dissimilarity, contrast, inverse difference, inverse difference moment and correlation [26].

### CNN-based features

**Fig. 3:**
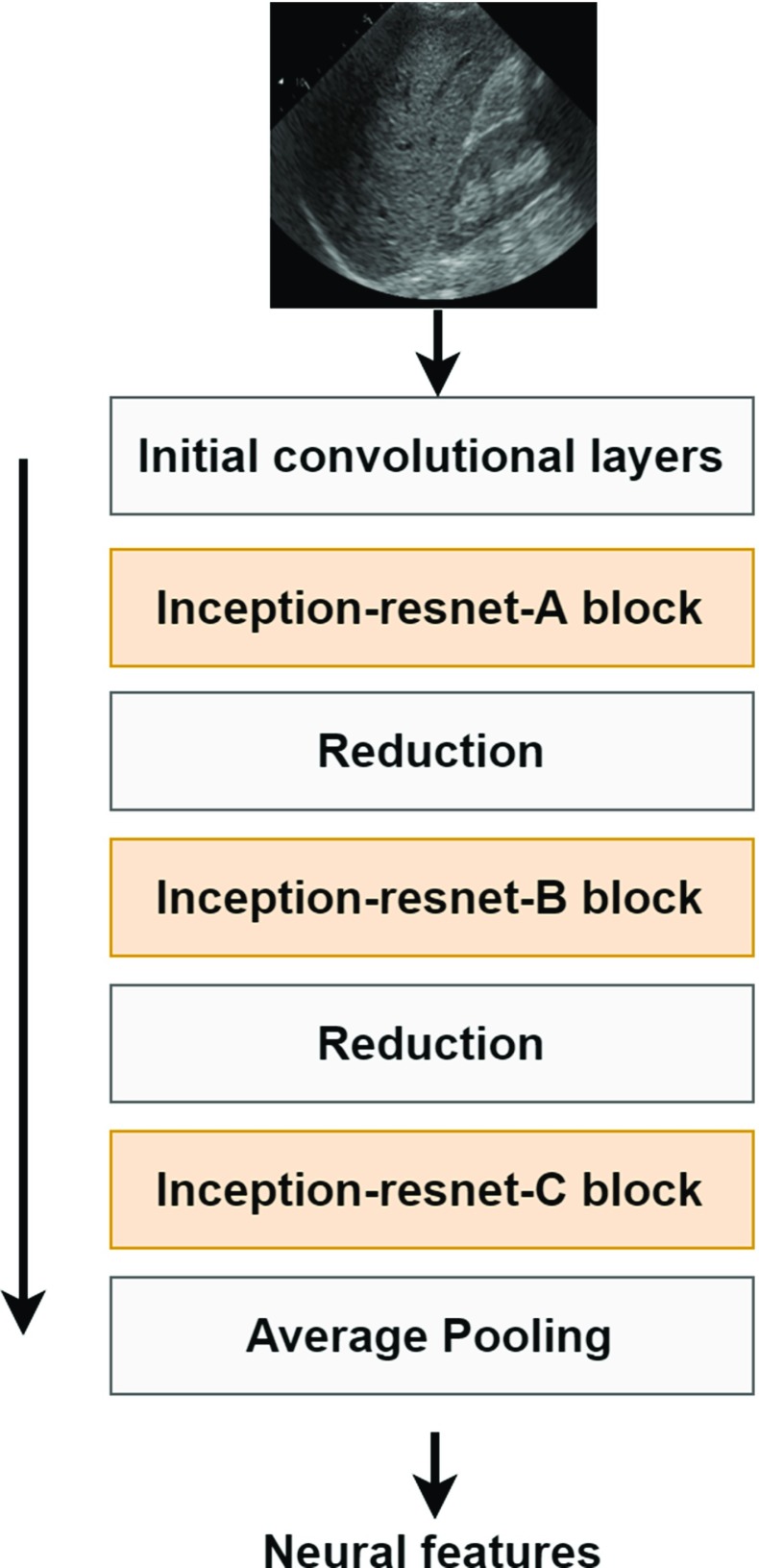
Illustration of feature extraction using the Inception-ResNet-v2 model [28]

CNN features were extracted using the Inception-ResNet-v2 CNN implemented in Keras [27, 28]. Calculations were performed in Python. The model was pre-trained on the ImageNet dataset [29]. This CNN includes a mixture of residual inception modules followed by grid reduction modules and has achieved state-of-the-art accuracy on the ImageNet dataset that contains 1.2 millions of labeled images [28]. Sample labels include animals, fruits and daily necessities. This dataset was successfully used for transfer learning in several medical imaging applications [16]. In our case, the CNN features were extracted using entire US images. Minimal pre-processing was applied to liver B-mode images, and the non-relevant data, such as frame number, were removed. Images were resized using the bi-cubic interpolation algorithm to the resolution originally designed for the network. Each liver image was given as the network input, and the corresponding neural features were extracted from the average pooling layer. Feature extraction procedure is depicted in Fig. 3. Next, zero-variance features were removed.

### Classification and evaluation

**Fig. 4:**
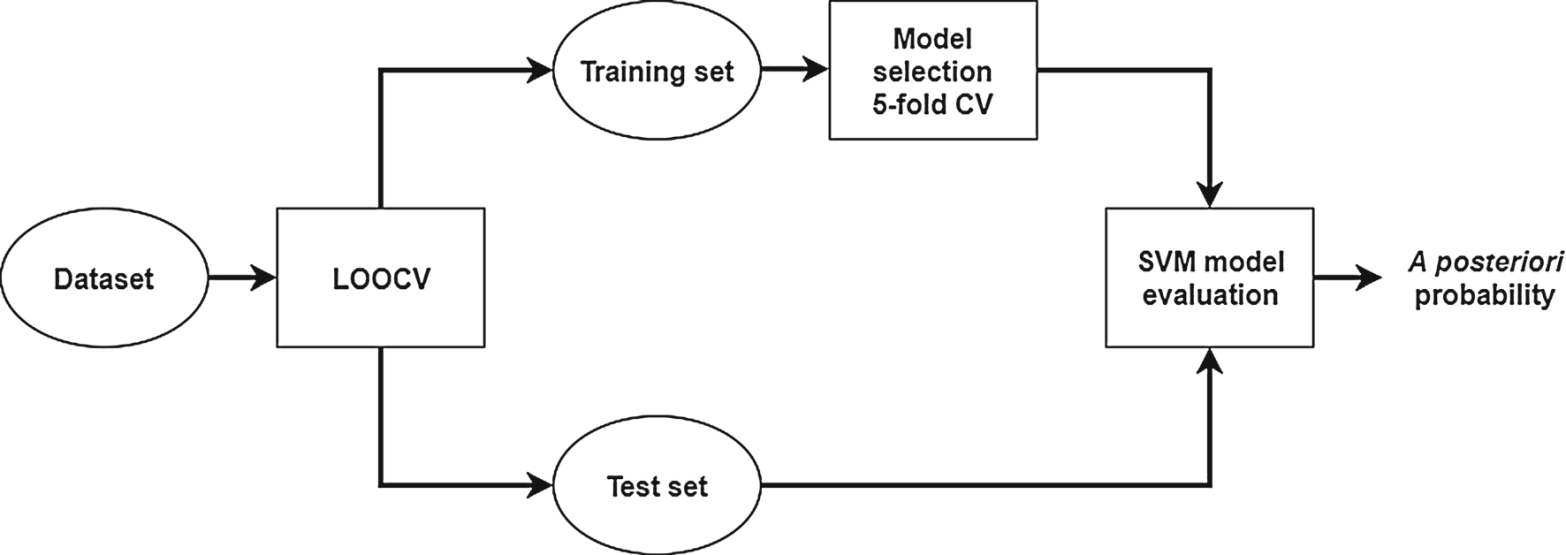
The validation pipeline

We utilized the SVM algorithm to perform the classification of fatty liver images [30] using the GLCM- or the CNN-based features. Methods that exclude outliers were used to normalize the features. The validation scheme is presented in Fig. 4. Patient-specific leave-one-out cross-validation (LOOCV) was applied to evaluate the classification. In each case, the test set consisted of 10 images from the same patient and the training set contained 540 images from the remaining 54 patients. For each training set, fivefold cross-validation and grid search were applied to indicate the optimal SVM classifier hyperparameters and the best kernel. To address the problem of class imbalance, the SVM hyperparameter *C* of each class was adjusted inversely proportional to that class frequency in the training set. Label 1 indicated the image containing a fatty liver and label − 1 otherwise. After the training phase, the a posteriori probabilities were calculated for each image in the test set and the results were averaged to obtain the final a posteriori probability related to the examined liver. Next, these probabilities were used to calculate the receiver operating characteristic (ROC) curve. The area under the ROC curve (AUC) was used for evaluation of the classification performance. We applied the Delong statistical test implemented in the pROC package in R to compare the AUC values obtained for all methods [31, 32].

To assess the level of steatosis, we employed the Lasso regression method. The same validation scheme was applied as in the case of the classification, but the steatosis level was estimated instead of the a posteriori class probability. Spearman correlation coefficients (SCCs) were calculated to assess the relation between the steatosis level, the models’ outputs and the HI parameter. Moreover, the SCCs between the models’ outputs and the HI parameter were determined. Next, the linear regression algorithm was used to relate the steatosis level and the HI parameter. All regression models were compared using the Meng test implemented in the cocor package in R [33, 34].

## Results

**Fig. 5:**
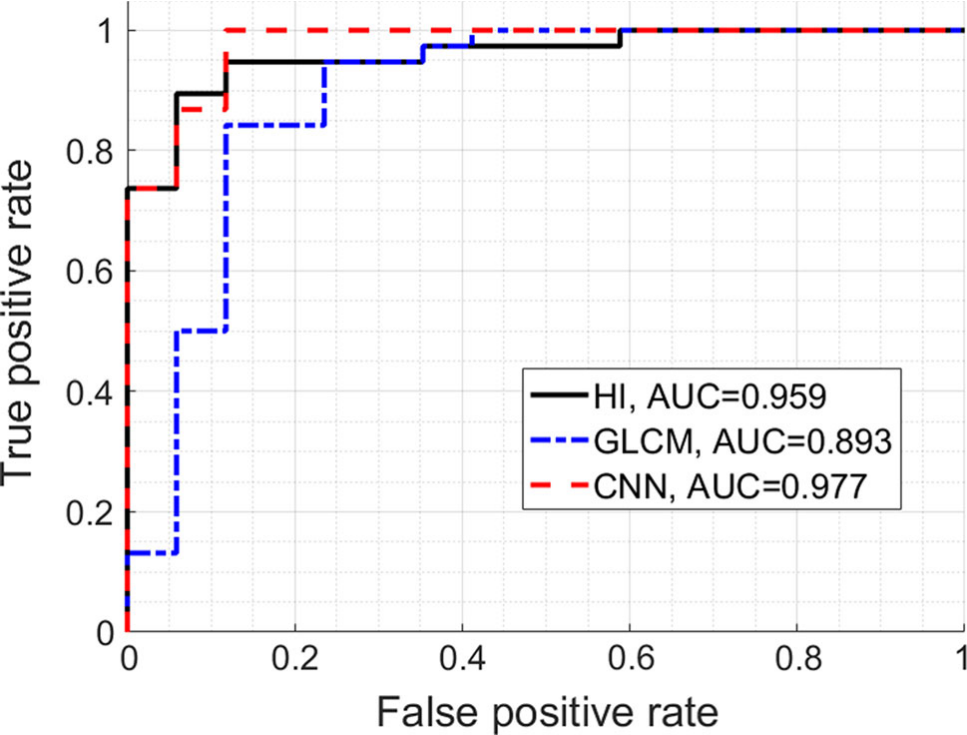
The ROC curves for the HI method (AUC = 0.959), the GLCM algorithm (AUC = 0.893) and the classifier developed using CNN features (AUC = 0.977)

The classification performance related to the HI parameter and the SVM classifiers is presented in Fig. 5. All methods achieved good classification performance. The highest AUC value, equal to 0.977, was obtained for the CNN-based classification. The performance of the HI-based approach was slightly lower, with AUC value equal to 0.959. However, the Delong test indicated that this difference in AUC values was not statistically significant (*p* value > 0.05). The AUC value obtained for the GLCM-based approach was equal to 0.892 and was significantly lower than that for the CNN-based method (Delong test *p* values < 0.05). The performance summary is presented in Table 1. Sensitivity, specificity and accuracy were calculated using the threshold corresponding to the ROC curve point, which was the closest to the upper left corner of the ROC plot, point (0, 1) [35].

**Table 1:** Classification performance summary

Method	AUC	Sensitivity	Specificity	Accuracy
Hepatorenal index	0.959 ± 0.044	0.895	0.941	0.909
GLCM	0.893 ± 0.059	0.842	0.882	0.854
CNN	0.977 ± 0.021	1	0.882	0.963

**Fig. 6:**
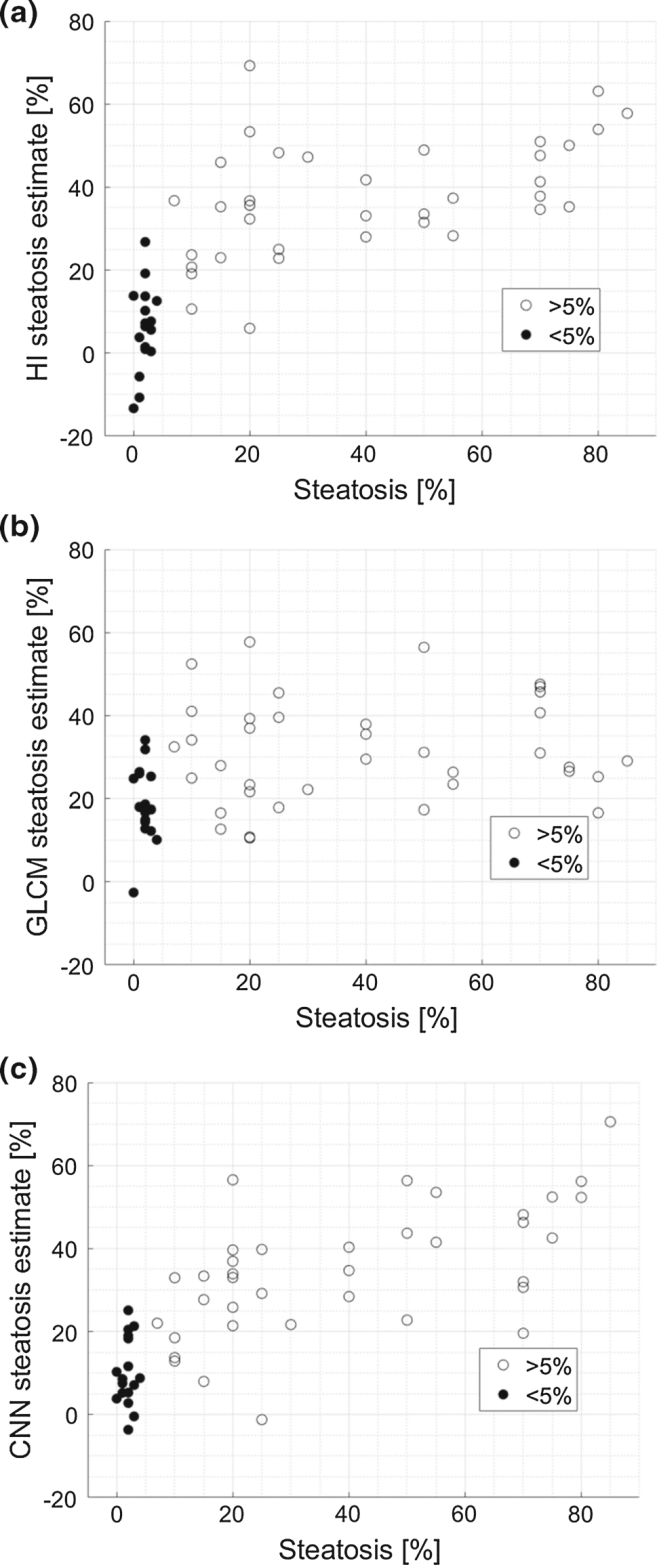
The usefulness of **a** the HI parameter (SCC = 0.80), **b** GLCM-based features (SCC = 0.39) and **c** the CNN-based features (SCC = 0.78) in steatosis level assessment

**Fig. 7:**
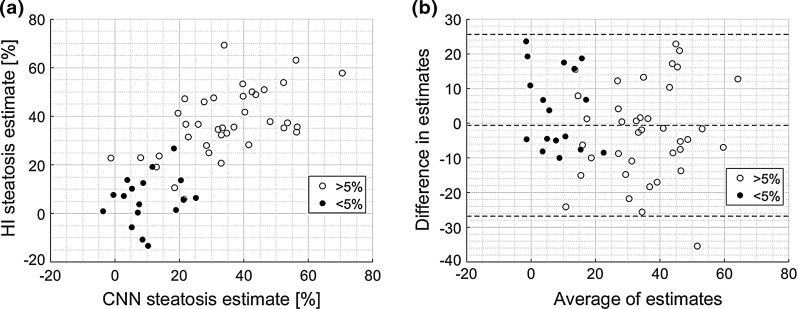
**a** The relation between the HI parameter and the Lasso regression (SCC = 0.78) and **b** the corresponding Bland–Altman plot

Figure 6 shows the usefulness of the Lasso regression method and the HI parameter in steatosis level assessment. The SCCs obtained for the Lasso algorithms utilizing the CNN- and the GLCM-based features were equal to 0.78 (*p* value < 0.001) and 0.39 (*p* value < 0.05), respectively. The SCC for the HI parameter was equal to 0.80 (*p* value < 0.05). The SCC between the CNN-based approach and the HI parameter was equal to 0.78 (*p* value < 0.05). The difference between the Lasso algorithm and the HI method correlation coefficients was statistically insignificant (*p* value > 0.05). Figure 7 illustrates the agreement between these two methods.

## Discussion

Ultrasound imaging is the most commonly applied imaging modality. Our study confirms that the HI parameter is a good predictor of steatosis level in liver. It is simple to calculate and efficient. Our results are in a good agreement with other studies reporting the usefulness of the HI parameter. We obtained high values of the AUC and the SCC parameters, which were equal to 0.959 and 0.80, respectively. The AUC values reported for the HI method ranges from 0.76 [36] to 0.99 [8]. However, the papers commonly report different ranges of the HI parameter and different optimal cutoffs for the fatty liver classification. [7, 8, 36–39]. This issue illustrates the ambiguity related to the HI-based fat assessment. The performance of the GLCM-based approach was worse with the AUC and the SCC equal to 0.893 and 0.39, respectively. Low value of the SCC parameter suggests that the GLCM-based features are not efficient for the steatosis level assessment. The obtained AUC value is in agreement with the results reported in the previous studies that employed GLCM-based features [12, 14, 15]. In [12, 15] the authors reported AUC values of around 0.8. In [14] the accuracy of around 0.8 was reported. In [13] the authors achieved high AUC value of 0.96. However, in this study the cross-validation was not applied and the authors used the same dataset to develop and evaluate the classifiers what could result in overfitting.

Our study shows the feasibility of using deep learning for the liver steatosis assessment. Although we used a small dataset containing only 550 images from 55 patients, these data were sufficient to develop a well performing classifier with transfer learning. The AUC value in the case of the fatty liver classification was equal to 0.977. According to Table 1, the obtained performance was higher than in the case of the HI method. Moreover, the CNN-based approach achieved significantly better results than the GLCM-based approach. The CNN features were useful and enabled efficient training of the classification and regression models. Good performance of the CNN-based approach was expected. In our study, we did not train the network from scratch, instead the pre-trained CNN was used for feature extraction. This model was developed using the ImageNet dataset containing 1.2 million labeled images of various objects. The HI calculation includes two convolutional operations (spatial averaging), which should be supposedly learned by the CNN to perform well on the ImageNet dataset. These two operations have to be conducted in the liver and the kidney, so the network has to detect these tissues first. The appearance of the liver with respect to surrounding tissues is important for efficient steatosis assessment.

In the case of the liver steatosis assessment, the obtained SCC, equal to 0.78, was slightly lower than the SCC calculated for the HI parameter, which was equal to 0.80. However, this difference was not statistically significant. Both regression models performed well, except for the patients with severe steatosis. In this case, the estimated values of steatosis were slightly too small. This may be due to the dataset, which was too small to build an accurate regression model. Moreover, the transfer learning in this case may not be efficient enough to capture the dependence between the input images and the liver steatosis level. Nevertheless, the proposed approach should be considered to be good, especially since the results were obtained in an automated process. Figure 7a illustrates the relation between the Lasso regression method and the HI parameter. In this case, the SCC was equal to 0.78, indicating high degree of correlation. According to the Bland–Altman plot in Fig. 7b, the average bias in estimates is low.

Although the performance of the proposed method was only slightly better than the performance obtained using the HI parameter, the proposed approach has several advantages that illustrate its clinical value. First, our method can be considered as an integrated computer-aided diagnosis system. It is operator independent and does not require ROI selection in comparison with the HI method and the GLCM-based approach. Next, the proposed method efficiently utilizes sequences of US images to assess the level of steatosis, while the approaches proposed in the literature commonly employs only one US image to conduct classification [6]. However, there are several issues related to our work. First of all, the ROI selection is operator dependent and has impact on calculation of the HI parameter and the GLCM-based features. For proper estimation of the HI parameter, the physician has to select ROIs in the liver and the kidney. These ROIs have to be as uniform as possible to omit the regions of blood vessels, ducts or other structures in the organs. In our study we focused on machine learning and did not examine observer variability, the ROIs were determined by a single physician. The obtained results may differ between observers [9, 10]. Second, all employed methods are to some extent scanner dependent. B-mode image intensities can be modified by using different image reconstruction and processing algorithms, what may affect the feature extraction and consequently the classification. This is a general issue encountered in studies that aim to develop US-based computer-aided diagnosis systems. Image quality (speckle patterns and boundary visibility) depends on scanner settings. The Inception-ResNet-v2 network utilized in our study was trained using the ImageNet dataset that contains images recorded under slightly different lighting conditions. Therefore, we believe that the impact of image reconstruction algorithms implemented in the US scanners should be lower for the proposed approach than in the case of the HI- and the GLCM-based methods. We would like to investigate this problem in the future in two ways. First, it would be interesting to acquire raw ultrasound data and investigate how the image reconstruction algorithms impact the feature extraction from the CNN [40]. Second, we are going to acquire B-mode images of the same liver using different scanner settings and investigate whether the model can learn features for classification that are independent of scanner settings. To make the assessment scanner independent, it would be interesting to employ the quantitative US techniques. These methods are used to estimate various physical properties of the tissue, such as the attenuation or scattering characteristics [41]. Quantitative US techniques can be used to create parametric maps that serve as an additional source of information on investigated tissue in comparison with standard B-mode images [42, 43]. Those maps may serve as a more proper input to the CNN than regular B-mode images. The usefulness of quantitative US techniques in liver steatosis assessment has been reported in several studies [13, 44, 45]. In the future, we plan also to acquire more data and investigate various approaches to model development.

## Conclusions

In this paper we proposed a CNN-based approach to steatosis level assessment utilizing B-mode ultrasound images. The model was developed using data acquired in obese patients undergoing wedge liver biopsy during bariatric surgery. Our approach is efficient and operator independent. Moreover, it outperforms the HI- and the GLCM-based classification.
